# An orally-available monovalent SMAC mimetic compound as a broad-spectrum antiviral

**DOI:** 10.1093/procel/pwad033

**Published:** 2023-06-09

**Authors:** Miao Mei, Maria_Antonietta Impagnatiello, Jun Jiao, Ulrich Reiser, Ulrike Tontsch-Grunt, Ju Zhang, Paul Nicklin, Bingke Yu, Yu Wang, Yuan He, Xu Tan

**Affiliations:** Tsinghua-Peking Center for Life Sciences, Beijing Advanced Innovation Center for Structural Biology, Beijing Frontier Research Center for Biological Structure, MOE Key Laboratory of Bioorganic Phosphorus Chemistry and Chemical Biology, School of Pharmaceutical Sciences, Tsinghua University, Beijing 100084, China; Chinese Institutes for Medical Research, Beijing 100069, China; Boehringer Ingelheim RCV GmbH & Co KG, Vienna, Austria; Tsinghua-Peking Center for Life Sciences, Beijing Advanced Innovation Center for Structural Biology, Beijing Frontier Research Center for Biological Structure, MOE Key Laboratory of Bioorganic Phosphorus Chemistry and Chemical Biology, School of Pharmaceutical Sciences, Tsinghua University, Beijing 100084, China; Chinese Institutes for Medical Research, Beijing 100069, China; Boehringer Ingelheim RCV GmbH & Co KG, Vienna, Austria; Boehringer Ingelheim RCV GmbH & Co KG, Vienna, Austria; College of Life Sciences and Oceanography, Shenzhen University, Shenzhen 518060, China; Research Beyond Borders, Boehringer Ingelheim, Biberach an der Riss, Germany; Research Beyond Borders, Boehringer Ingelheim, Shanghai 200120, China; College of Life Sciences and Oceanography, Shenzhen University, Shenzhen 518060, China; Research Beyond Borders, Boehringer Ingelheim, Shanghai 200120, China; Tsinghua-Peking Center for Life Sciences, Beijing Advanced Innovation Center for Structural Biology, Beijing Frontier Research Center for Biological Structure, MOE Key Laboratory of Bioorganic Phosphorus Chemistry and Chemical Biology, School of Pharmaceutical Sciences, Tsinghua University, Beijing 100084, China; Chinese Institutes for Medical Research, Beijing 100069, China

## Dear Editor,

The coronavirus disease 2019 (COVID-19) pandemic caused great global morbidity and mortality concurrent with astronomical socioeconomic damages and losses. Until now, the culpable pathogen, SARS-CoV-2, has infected over 6 hundred million people resulting in more than 6 million deaths worldwide (WHO SARS-CoV-2 cases). There is an urgent need for rapid development of effective and affordable therapeutics for COVID-19 patients. However, an average of 12 years is required from target discovery to approval of a safe and selective antiviral agent (Chitalia and Munawar, 2020), not meeting the expectations of the society. Therefore, there is a great unmet medical need for broadly acting antiviral therapeutics to treat multiple viruses (Mei and Tan, 2021), which can be rapidly repurposed up to the speed of the emergence and circulation of pathogenic viruses. SMAC mimetic is a class of agents which mimic endogenous SMAC protein to inhibit Inhibitor of apoptosis proteins (IAP) family protein functions (Bai et al., 2014). IAPs function as ubiquitin E3 ligases and inhibit TNFα-mediated apoptotic pathway. When TNF-α binds to its receptor, TNFR1, IAP family members cellular IAP1 (cIAP1) and cellular IAP2 (cIAP2) are recruited and facilitate activation of NF-κB and MAP kinases, resulting in cytokine release and cell survival (Ladner et al., 1997; Xiang et al., 2019). SMAC mimetics, which can be monovalent or bivalent referring to the stoichiometry of binding one or two IAPs, were developed to induce immunogenic cell death of tumor cells (Petersen et al., 2007; Bai et al., 2014). Currently, several compounds have entered clinical trials for cancer treatment. As for antiviral therapy, research focuses on SMAC mimetic for treatment of chronic viral infections, such as Hepatitis B virus (HBV) and Human Immunodeficiency Virus (HIV) (Ebert et al., 2015; Pache et al., 2015, 2020). Two bivalent SMAC mimetics, birinapant and APG-1387 entered clinical trials for HBV treatment (NCT02288208, NCT03585322). Birinapant was terminated in clinical phase I trial due to adverse effect including an association with Bell’s Palsy. Compared with reported bivalent ones, monovalent compounds might be advantageous for their oral bioavailability and safety/tolerability profile. However, in previous reports monovalent compounds showed limited *in vivo* efficacy against HBV infection (Ebert et al., 2015). Considering the great unmet medical need of a functional cure of chronic HBV infection, continuous efforts are justified to develop new monovalent SMAC mimetic compounds for HBV treatment. Here we report a potent monovalent SMAC mimetic BI-82 with novel chemical structure. In this study, we investigated the antiviral effect of BI-82 in a chronic HBV infection model which delivers rcccDNA to the liver through a recombinant adenoviral vector. Our results show that BI-82 has a long-term efficacy decreasing HBV antigen levels, with a reduction of liver inflammation and fibrosis during late stages of viral persistence. The results show that the antiviral effects of orally given BI-82 is comparable to those of intraperitoneal treated birinapant. Besides chronic HBV infection, we also investigated BI-82’s antiviral potential in acute viral infections including *flaviviruses*, SARS-CoV-2 and influenza A virus (IAV). We found that BI-82 showed a strong potency against SARS-CoV-2 Omicron variant *in vitro* and efficacy against influenza infection *in vivo*. In conclusion, our work identifies a novel SMAC mimetic compound BI-82 as a broad-spectrum antiviral with potential of treating both acute and chronic viral infections.

**Figure 1.**
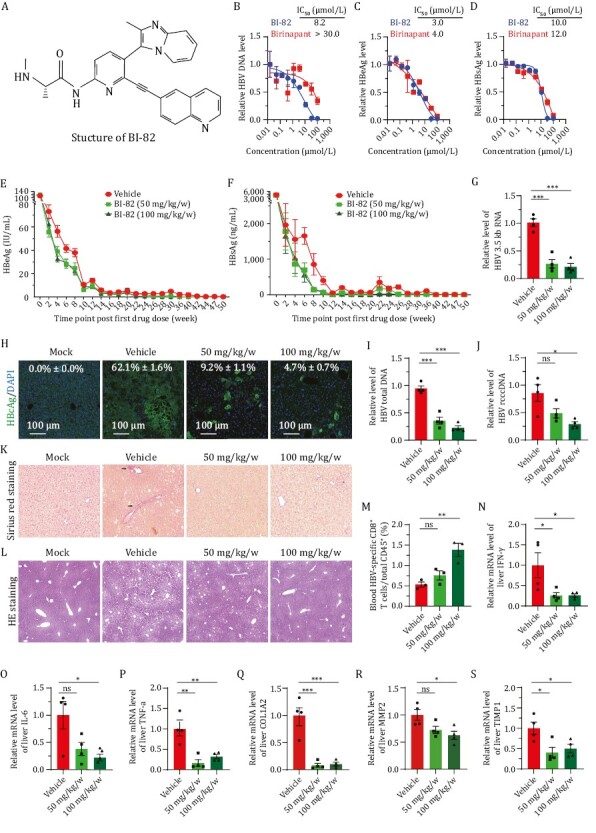
**SMAC Mimetic BI-82 attenuates HBV infection *in vitro* and *in vivo*.** (A) Structure of BI-82 and a cartoon scheme showing the mechanism of action of SMAC Mimetics. TNF-α interaction with TNFR1 and TNFR2 elicits either apoptosis by recruiting different adaptor proteins or NIK/TNF-α inflammation/survival pathway. The extrinsic pathway is triggered by binding of death receptors with death ligands (i.e., TNF-α, FasL, TRIAL, etc.) followed by formation of the death-inducing signaling complex, activation of caspase 8 and transmission of death signals to effector caspase, leading to apoptotic cell death. The intrinsic pathway is induced by a number of factors, including DNA damage to initiate the apoptotic cascade of death signals through interaction with specific downstream mediator of  apoptosis. Then  the formation of mitochondrial pores and  change of membrane permeability release SMACs  and cytochrome c into the cytoplasm to facilitate the activation of downstream apoptotic signal (caspase 9, 3, or 7), eventually resulting in apoptosis. Highly expressed IAPs inhibit apoptotic signaling cascades in tumor cells and infected cells with virus. SMAC Mimetics like BI-82, can specifically suppress IAPs protein levels to induce cell apoptosis. TNF, tumor necrosis factor; TRAIL, TNF related apoptosis inducing ligand; tBID, truncated BH3 interacting-domain death agonist; cIAP, cellular Inhibitors of apoptosis; XIAP, X-linked inhibitor of apoptosis. (B–D) Primary human hepatocytes (PHH) were treated with birinapant and BI-82, and infected with HBV for 6 days, then supernatants and cells were harvested to test viral titer through qRT-PCR for HBV DNA (B) and ELISA for HBs/eAg (C and D), *n* = 3. (E and F) BI-82 markedly inhibits HBeAg (E) and HBsAg (F) expression by ELISA in a persistent HBV mouse model. In 8–10 weeks old Alb-Cre male mice, 1.5 × 10^9^ PFU of Ad-HBV was injected by intravenously administration. Next day, sera HBV antigens were quantified by ELISA for grouping infected mice with equal HBV titer. At 2 dpi, BI-82 were delivered by weekly oral administration for 16 weeks, *n* = 11. (G, I and J) BI-82 decreased intrahepatic RNA (G), HBV total DNA (I), and rcccDNA (J) level as measured by qRT-PCR at week 11 after administration. (H) BI-82 decreased intrahepatic HBcAg expression by immunofluorescence staining at week 11 after administration. (K) BI-82 protected liver from fibrosis induced by HBV as measured by Sirius red staining, black arrows indicate collagen fibers. (L) BI-82 protected the integrity of liver structure shown by HE staining. (M) BI-82 alleviated HBV-specific CD8^+^ T cell depletion. At Week 24, mice blood cells were stained with anti-mCD15, anti-mCD8 and anti-HBV-core tetramers, the HBV-specific CD8^+^ T cells were analyzed via FACS. (N–P) BI-82 attenuated HBV-induced intrahepatic inflammatory factors expression measured by qRT-PCR, including IFN-γ (N), IL-6 (O) and TNF-α (P). (Q–S) BI-82 attenuated HBV-induced intrahepatic fibrosis markers expression measured by qRT-PCR, including COL1A2 (Q), MMP2 (R), TIMP1 (S).

The SMAC mimetic BI-82 is a 2-amino-*N*-(6-ethynylpyridin-2-yl) propenamide synthesized by Boehringer Ingelheim (detailed description in patent WO 2013/127729) (Fig. 1A). It potently inhibits cIAPs with selectivity for cIAP1 and cIAP2 vs. XIAP. It binds to the BIR3 domain of cIAP1 with an IC_50_ of 0.90 ± 0.15 nmol/L, cIAP2 with an IC_50_ of 17.10 ± 5.37 nmol/L and XIAP with an IC_50_ of 273.3 ± 82.46 nmol/L. BI-82 inhibits cIAP1/2 and XIAP to promote apoptosis particularly in infected cells due to the heightened inflammatory pathways such as the TNF-α signaling pathway during infection (Fig. 1A). We first investigated BI-82’s potential effect on HBV replication *in vitro* by using a recombinant cccDNA (rcccDNA) cell model described previously (Li et al., 2018). Briefly, monomeric HBV genome bearing a loxP-chimeric intron was delivered by adenovirus into human hepatoma Huh7 cells stably expressing the Cre recombinase. Cre/loxP-mediated site-specific recombination and subsequent RNA splicing will generate rcccDNA, which allows expression and replication of HBV in the cell line. We infected Huh7-Cre cell line with Adv-rcccDNA at an MOI of 50 and treated the cells with different concentrations of BI-82 at the same time. At 72 h post transfection, significant decrease of HBsAg (IC_50_ ≈ 10 nmol/L), HBeAg (IC_50_ ≈ 25 nmol/L), HBV rcccDNA (IC_50_ ≈ 14 nmol/L) and HBV RNA (IC_50_ ≈ 296.5 nmol/L) was observed (Fig. S1A–D). This effect is dose-dependent, and the potency is comparable to that of birinapant, the bivalent SMAC mimetic compound which entered clinical phase I trial for HBV treatment. These data indicated that BI-82 can suppress rcccDNA-driven transcription and/or replication. To further determine the effect of BI-82 on HBV, we used well-established primary human hepatocyte (PHH) model. One day after the infection of PHH with HBV, cells were treated with BI-82 and birinapant at different concentrations. HBsAg, HBeAg, and HBV DNA levels in the supernatant were measured 6 days after treatment. Consistent with the data of rcccDNA assay, BI-82 treatment significantly decreased HBV antigen levels, including both HBsAg and HBeAg, as well as HBV DNA level in the supernatant of HBV-infected PHH (Fig. 1B–D). In the PHH assay, SMACs antiviral activity requires much higher concentration compared to Ad-rcccDNA assay, which might be due to the higher infection rate in the Ad-rcccDNA assay that would generate more cells susceptible to apoptosis. We next tested whether BI-82 inhibits HBV infection by inducing apoptosis. We transduced Huh7-Cre with Ad-rcccDNA vector for 3 days, then trypsinized and reseeded cells into a 24-well plate and added compounds to treat for another 2 days. Following staining with anti-HBcAg and TUNEL reagent demonstrated that BI-82 and Birinapant stimulate apoptosis of HBV-infected cells compared with DMSO control (Fig. S1E). These results indicated that BI-82 inhibits HBV replication by inducing apoptosis *in vitro*.

For *in vivo* study, we first tested safe concentration range of orally administration of BI-82 in wildtype C57BL/6J mice and foundless than 200 mg/kg/0.5 w is safe without weight loss for 4 weeks. Our mouse pharmacokinetic data show that oral administration of BI-82 can lead to a maximum serum concentration of BI-82 as high as 5.5 μmol/L (± 1.1 μmol/L) without causing serious *in vivo* toxicity. Its half-life in serum is 3.0 h (± 1.1 h), indicating a reasonable drug stability. We next investigated BI-82 in a hydrodynamic acute mouse infection model (Yang et al., 2002). In this model HBV infection lasts for about 1 week. Oral administration of BI-82 twice weekly significantly decreased HBsAg and HBeAg in mouse serum in a dose-dependent fashion (Fig. S2A–C). We included intraperitoneal administration of birinapant as a control and the efficacy of BI-82 was comparable to that of birinapant, which has been reported earlier (Ebert et al., 2015). To further investigate the effect of BI-82 in chronic infection, we used a rcccDNA mouse model which closely mimics progressive pathology of clinical chronic hepatitis in human as previously described (Li et al., 2018). 1.5 × 10^9^ PFU of Ad-rcccDNA was injected into Alb-Cre Tg mice via tail vein and BI-82 was administered with two dosing regimens (50 mg/kg/w and 100 mg/kg/w) continuously for 16 weeks. HBsAg and HBeAg were monitored up to 50 weeks post infection and a clear decrease of HBsAg and HBeAg levels in mouse sera was observed (Fig. 1E and 1F). This effect was maintained for the whole experiment duration. SMAC mimetics are known to promote ubiquitination and proteasomal degradation of cIAP1, which is considered the major target of anti-HBV effect. We examined cIAP1 and cIAP2 levels in liver by Western blot at week 11. A reduction of cIAP1 but not cIAP2 in the liver was observed (Fig. S3A and S3B), consistent with the on-target effects of SMAC mimetics for HBV treatment reported in previous reports (Ebert et al., 2015). As consequences of antagonizing cIAP1, the HBV core antigen (HBcAg) in liver tissue also showed a much lower level by immunohistochemical staining in BI-82 treatment groups at week 11 (Fig. 1H). The levels of HBV RNA, DNA and cccDNA in mouse liver all showed significant reductions at week 11 (Fig. 1G, 1I and 1J). Compared with birinapant administered intraperitoneally (30 mg/kg/w), orally given BI-82 (100 mg/kg/w) has a largely comparable efficacy against chronic HBV infection (Fig. S4A–C). Structure-wise, sinus red staining of liver tissue showed that after BI-82 treatment, mice were prevented from liver scarring in the portal areas which indicates liver fibrosis progression (Figs. 1K and S3C). In the meantime, injury to the integrity of liver tissue was attenuated in BI-82 treatment group as measured by HE staining (Figs. 1L and S3D).

Patients with chronic HBV infection are characterized by an exhausted CD8 T cell response (Maini et al., 2000). Our results showed that 100 mg/kg/w of BI-82 significantly improved HBV-specific CD8 T cell responses at Week 11 (Figs. 1M and S3E). Besides the inhibitory effect of BI-82 on viral replication, we investigated whether BI-82 alleviates HBV-associated pathology. The chronic rcccDNA model was reported to be analogous to the progressive pathology of clinical chronic hepatitis with a sustained inflammatory response, fibrosis and dysplastic lesions commonly seen during late stages of viral persistence (Li et al., 2018). We evaluated BI-82’s effect on HBV-induced inflammation and liver fibrosis at late stages of infection (Week 24). We first examined the intrahepatic levels of inflammatory factors such as IFN-γ, IL-6, TNF-α by qRT-PCR and significantly reduced levels of these factors were observed (Fig. 1N–P) at Week 24. The qRT-PCR data also showed that BI-82 can significantly downregulate the expression levels of fibrosis-related genes in the liver tissue, including alpha-2 type 1 collagen (Col1a2), matrix metallopeptidase 2 (MMP2) and tissue inhibitor of metalloproteinase-1 (TIMP1) (Fig. 1Q–S).

**Figure 2.**
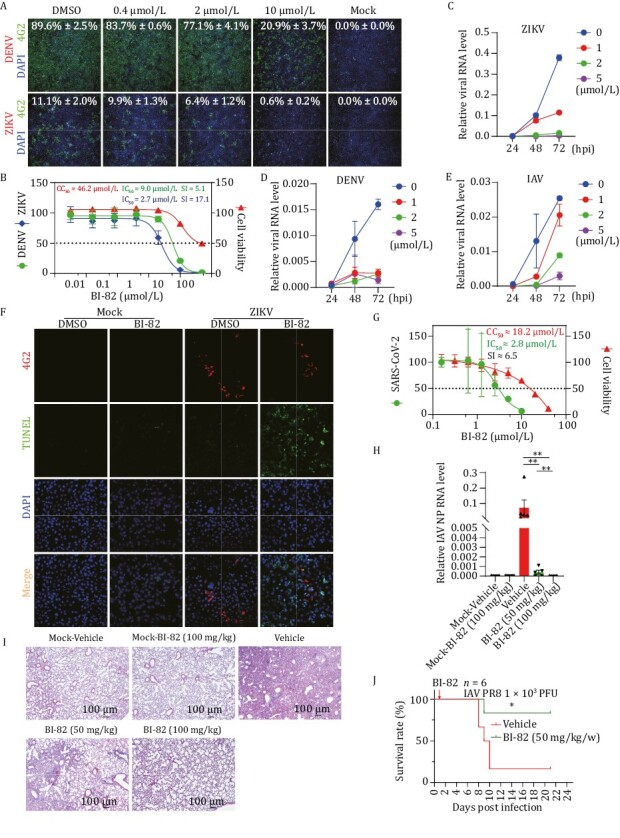
**BI-82 inhibits RNA virus infection *in vitro* and *in vivo*.** (A and B) BI-82 inhibits DENV and ZIKV infection *in vitro*. BI-82 and viruses were concurrently added into Vero cells with 1.0 MOI DENV or 0.2 MOI ZIKV strain for 48 h, and stained with anti-flavivirus group antigen (4G2), the infection ratios and cell numbers were analyzed by high content microscope. (C–E) BI-82 influences the multi-step growth kinetics of multiple virus infections. Vero and Hela cells were seeded into 12-well plates one day before being treated with 5 µmol/L BI-82 or DMSO and infected concurrently with 0.2 MOI of ZIKV (Vero), 0.05 MOI of DENV (Vero) and 0.5 MOI IAV. Then equal volume of the supernatant was harvested separately at 24, 48 and 72 hpi, and used to infect Vero or Hela cells with as a second-round infection for 48 h, the viral RNA level was analyzed through qRT-PCR. (F) BI-82 increases apoptosis ofinfected cells with ZIKV. Vero cells were seeded into 24-well plate one day before being treated with 5 µmol/L BI-82 or DMSO and infected concurrently with 0.2 MOI of ZIKV for 48 h. The cells were stained with anti-4G2 and TUNEL kit and scanned with confocal microscope. (G) BI-82 inhibits SARS-CoV-2 infection in Vero cells . Vero cells were treated by BI-82 and infected with 200 TCID_50_/100 μL for 2 days, supernatant from infected Vero cells were titrated by qRT-PCR. (H) BI-82 significantly decreases IAV load in the lungs 7 days post infection as measured by qRT-PCR (*n* = 5). (I) BI-82 alleviates IAV induced histopathological lesion of the lungs 7 days post infection as measured by HE staining (*n* = 5) (scale bar =100 µm). (J) BI-82 inhibits influenza A virus infection in C57/BL6J mice. Eight to ten weeks old mice were infected with 10^3^ pfu of IAV by nasal drop infection and treated with BI-82 (50 mg/kg/w) at 24 hpi and the body weights were recorded daily up to 21 days to evaluate the survival curve (*n* = 6).

A number of viruses have been reported to be associated with increased apoptosis, including *flaviviruses*, influenza virus, SARS-CoV-2, so targeting virus-induced apoptosis could be a promising strategy in the treatment of acute virus infections. Host-directed agents have advantages over direct antivirals for their broad-spectrum antiviral potential and a possibly higher barrier to drug resistance (Mei and Tan, 2021). IAP antagonists previously have been shown with efficacy in several chronic viral infections such as HBV and HIV (Ebert et al., 2015; Clark et al., 2021; Molyer et al., 2021). We aim to test additional antiviral applications of SMAC mimetics. First, we evaluated BI-82 in flaviviruses (DENV and ZIKV) *in vitro*. We concurrently added BI-82 and DENV (Dengue-2 New Guinea C strain) and ZIKV (GZ-01 strain) into Vero cells for 48 h. The results show that BI-82 can broadly inhibit DENV with an IC_50_ (the concentrations required to inhibit 50% of virus proliferation) of 9.0 µmol/L and a selectivity index of 5.1, and ZIKV infection with an IC_50_ of 2.7 µmol/L and a selectivity index of 17.1 (Fig. 2A and 2B) (Selectivity index is CC_50_/IC_50_, CC_50_ is the concentration causing the loss of 50% of cell viability). We evaluated the effect BI-82 on multi-step growth kinetics of ZIKV (Fig. 2C), DENV (Fig. 2D) and IAV (H1N1 A/PR/8/1934 strain)(Fig. 2E) with a time course experiments in which we harvested the supernatants from the infected cells at 24, 48 and 72 h post infection and used these supernatants to performed a second round of infection to measure the infectivity of progeny virions. The results show that BI-82 significantly reduced the production of progeny virions. By TUNEL staining, BI-82’s antiviral effect is correlated with its promoting apoptosis of infected cells (Figs. 2F, S5A and S5B). Furthermore, we found that BI-82 can also inhibit acute viral infections including SARS-CoV-2 and influenza A virus (Fig. 2G and 2H). We evaluated and quantified the effect of BI-82 on secreted viral RNA of SARS-CoV-2 in the cell supernatant. Vero cells were infected with 200 TCID_50_/100 µL SARS-CoV-2 virus and treated with different doses of BI-82. We quantified the secreted viral RNA of SARS-CoV-2 in the cell supernatant by qRT-PCR and found a potent *in vitro* inhibition effect. The IC_50_ of virus proliferation is 2.82 µmol/L and the CC_50_ is 18.2 µmol/L, with a seletivity index around 6.5-fold indicating a potential therapeutic window (Fig. 2G). In order to further confirm our finding *in vivo*, we evaluated BI-82 in influenza A virus infection in a mouse model. Our data showed that oral administration of BI-82 protected mice from lethal challenge of IAV (Fig. 2H–J). Two doses of 50 mg/kg or 100 mg/kg BI-82 significantly decreased viral load in the lung (Fig. 2H) and prevented lung histopathological lesion from 1 × 10^3^ PFU of IAV (Fig. 2I). Meanwhile, BI-82 administration also decreased gene expression of inflammatory cytokines and interferon-stimulating genes in lung (Fig. S6A–G). One dose of 50 mg/kg BI-82, administered after influenza A virus challenge, resulted in a significantly increased survival in mice (80% survival on Day 21 post infection compared with lower than 20% survival in the mock-treated group) (Fig. 2J). BI-82 mediated protection correlates with reduced viral load and reduced cytokine induced pathology. These data support the potential broad-spectrum efficacy of BI-82 in treating both chronic and acute viral infections.

Previously cIAPs were reported to act as restriction factors preventing TNFα-mediated elimination of HBV. It followed that SMAC mimetics antagonizing cIAPs were developed for HBV treatment, resulting in bivalent compounds entering clinical trials. There is conflicting preclinical efficacy data for monovalent compounds and no monovalent SMAC mimetic is under clinical trial for HBV treatment. Here we showed that a well-tolerated orally administered monovalent IAP inhibitor, BI-82, is able to inhibit HBV replication both *in vitro* and *in vivo* by inducing apoptosis of infected cells. With a completely new chemical structure, BI-82 represents a novel class of SMAC mimetic with excellent bioavailability, safety and efficacy for antiviral development.

A functional cure for HBV is difficult to achieve partially due to the extremely stable cccDNA pool. cccDNA exists as an episomal mini-chromosome in the nucleus of hepatocytes and is resistant to sustained liver inflammation and antiviral treatments. It is considered as a primary molecular mechanism for HBV persistence. Previous reports showed that targeting cIAPs induces a preferential apoptosis of HBV-infected hepatocytes. We show that the orally-available SMAC mimetic BI-82 is capable of eliminating cccDNA in the liver, which brings the hope of achieving a functional cure of chronic HBV infection. In this study, we observed a rapid drop of HBeAg and HBsAg in the early infection stage (earlier than 8 weeks post infection) even in mice receiving vehicle treatment, including the previous report (Li et al., 2018). A possible explanation is that the quick formation of rcccDNA via Cre/loxP system induce an acute infection-immune response, Ad-rcccDNA vector was partially eliminated by the induced immune response. Nevertheless, the infection quickly induced apoptosis and BI-82 further triggered the deaths of infected cells. In addition, our results showed that BI-82 elevates the population of HBV-specific CD8^+^ T cell, which might be due to the effect of Smac mimetics on reinvigorating exhausted CD8^+^ T cells or an enhanced presentation of HBV antigens after the apoptosis of infected cells. Clinically, HBV infection is responsible for inducinghepatic fibrogenesis, which ultimately leads to cirrhosis. The Ad-rcccDNA mouse model was reported to closely resemble clinical chronic viral hepatitis with progressive liver pathology and liver fibrosis. We investigated BI-82 in this mouse model and found that BI-82 can ameliorate the severity of liver injury and fibrosis. Our results further showed that BI-82 decreased inflammatory cytokines, potentially leading to the attenuated fibrosis observed. This therapeutical effect via targeting the host cells is synergistic with the currently used direct antivirals such as entecavir and therefore justifies BI-82 as part of a promising combinatorial therapy.

We also first report that a SMAC mimetic compound BI-82 has efficacy against acute viral infections including Dengue virus, Zika virus, the Omicron variant of SARS-CoV-2 and influenza A virus. It follows that BI-82 might be also efficacious against multiple other viruses and that other SMAC mimetic compounds might have antiviral potentials, which warrant intense future investigations.

## Supplementary Material

pwad033_suppl_Supplementary_Materials

## Footnotes

This work was supported by grants from the State Key Research Development Program of China to X.T. (2022YFE0102200 and 2021YFC2300200), the Spring Breeze Fund of Tsinghua University and funding from Boehringer Ingelheim International GmbH. M.M. was supported by a grant from the National Natural Science Foundation of China to M.M. (32100107), the fellowship of China Postdoctoral Science Foundation (2021M691807) and postdoctoral fellowships from Tsinghua-Peking Life Center. We thank Kuanhui Xiang (Peking University) for PHH and Qiang Deng (Fudan University) for Ad-HBV vector.

I.M.A, R.U., T.G.U, N.P and Y.B. and Y.H. are employees of Boehringer Ingelheim.

Animal experiments were approved by governmental Institutional Animal Care and Use Committee (IACUC), Office of Laboratory Animal Welfare (OLAW) and Tsinghua Animal Ethics Committee.

X.T. and Y.H conceived and supervised the project. M.M. conducted most of the experiments. I.M.A performed *in vitro* characterization of BI-82. J.J. performed HBV infection in PHH. R.U. generated the compound. T.G.U, N.P and Y.B.K performed data analysis and scientific discussions. J.Z. and Y.W. conducted experiments on SARS-CoV-2 infection. Y.H. and X.T. wrote the manuscript.

Authors declared no conflict of interest.
