# Supplementary material for: An orally-available monovalent SMAC mimetic compound as a broad-spectrum antiviral
Source: Protein Cell. 2023 Jun 9;15(1):69–75. doi: 10.1093/procel/pwad033 (PMC10762662; doi:10.1093/procel/pwad033)
Supplement: pwad033_suppl_Supplementary_Materials [file pwad033_suppl_supplementary_materials.pdf]

An orally available monovalent SMAC mimetic compound as a broad-spectrum antiviral

Miao Mei[1,2*], Maria_Antonietta Impagnatiello[3*], Jun Jiao[1,2], Ulrich Reiser[3], Ulrike Tontsch-Grunt[3], Ju Zhang[4], Paul Nicklin[5], Bingke Yu[6], Yu Wang[4], Yuan He[6#], Xu Tan [1,2#]

This file includes:

Materials and methods

Figure S1-S7 and legends

Materials and Methods

Cells and cell culture

The Huh-7, Hep G2, HEK293 and HEK293T cells were cultured in Gibco DMEM medium supplemented with 10% heat inactivated fetal bovine serum (FBS) (PAN Biotech), 1% GlutaMX (Gibco), 1% penicillin/streptomycin (Gibco) in an incubator at 37 ℃with 5% CO_2. Huh7-Cre cells were cultured in complete DMEM with 1 μg/ml purimycin. Primary human hepatocytes (PHH) were kindly provided by Prof. Kuanhui Xiang, Peking University Health Science Center. PHH were seeded in 24-well plate coated with 5 mg/ml collagen type I (Gibco, A10483) and maintained in 5C media [5].

Recombinant Ad-HBV viruses

Recombinant Ad-HBV (mono) vector is by a courtesy of Prof. Qiang Deng from Fudan University [10]. The protocol for virus production and amplification is as follows [10, 15]: the vector is linearized with *Pac*I and transfected into HEK293 cells with PEI to amplify recombinant Ad-HBV virus. The viruses were purified via cesium chloride gradient centrifugation and the viral titer was titrated by standard plaque assay.

ccHBV production

Cell culture-derived HBV (ccHBV) viral stocks for PHH infection were obtained from the culture medium of HepAD38 cells. HepAD38 cells were cultured with Dulbecco's modified Eagle medium supplemented with 10% fetal bovine serum, and supernatant was collected every two days. Infectious HBV particles were concentrated from culture supernatants by precipitation with 5% PEG8000 (Sigma, 89510-1KG-F) and then resuspended in 5C media. Resuspended HBV viruses were mixed well overnight with 4% PEG8000. The viral titer (genome equivalent, GE) was determined by a quantitative RT-PCR assay.

SARS-CoV-2 Omicron BA.2 infection

Vero cells (8,000) were seeded into a 96-well plate and pretreated with BI-82 of different concentrations for 1 hours, then infected with 100 $TCID_{50}$ SARS-CoV-2 Omicron BA.2 for 96 hours. The supernatant of each treatment was titrated through qRT-PCR assay, the relative viral titer is normalized to DMSO control group.

Mouse experiments

All mice were maintained under pathogen-free conditions and used in accordance of governmental Institutional Animal Care and Use Committee (IACUC), Office of Laboratory Animal Welfare (OLAW) and Tsinghua University guidelines for animal welfare (Tsinghua University Assurance Identification No. F16-00228, A5916-01). The animal study protocol was approved by Tsinghua Animal Ethics Committee (AP#: 18-TX1 and date of approval: 20180929). For hydrodynamic injection (HDI), wild-type male C57BL/6 mice aged at 6-8 weeks were weighed and hydrodynamically injected with 10 μg pHBV1.3x ayw each in a volume of PBS determined by 1 ml/10g body weight. After 24 hours, blood was collected, and mice were grouped with HBV antigen titer quantified by ELISA. The BI-82 formation for oral dosing in mice is 10.55 mg/ml containing 10.55 mg BI-82 (MW 460) in ethanol (100 mg/ml), 91.6 μl 1M Citric acid and 0.5% natrasol (2.5 g natrasol was resolved completely in ddH_2O through stirring and heating to transparent) in 1ml solution. At 48 hpi, BI-82 and birinapant (30 mg/kg, in saline solution) administrated on Day 0 and Day 3 by gavage and intraperitoneal injection (i.p.), respectively, and entecavir (3.2 mg/kg/d) were resolved in peanut oil and administrated through gavage each day from Day 0 to Day 5. On day 6, blood was collected and sera HBV antigens were measured by ELISA. For Ad-HBV infection, in 8-10 weeks old male Alb-Cre mice (Jackson lab strain #003574), $1.5x10^9$ PFU of Ad-HBV was injected by intravenously administration. Next day, sera HBV antigens were quantified by ELISA for grouping infected mice with equal HBV titer. At 2 wpi, BI-82 (50 mg/kg/w, 100 mg/kg/w) was delivered by consecutively oral administration weekly for 16 weeks. Birinapant (30 mg/kg/w) was administrated by i.p. weekly and Entecavir was

delivered by oral administration (3.2 mg/kg/d). The mice were euthanized at time points specified in the main text, blood was harvested for ELISA measurement and liver samples for qRT-PCR, immunoblotting and tissue anti-HBcAg staining analysis. For IAV infection, mature male mice were nasally infected with a titer of 10^3 PFU of IAV, BI-82 (50 mg/kg) was orally administrated at 24 hours post infection and 0.5% natrasol as vehicle control. The lung sample were collected for H.E staining and qRT-PCR analysis on Day 7. The body weight of mice infected with IAV was recorded to calculate the survival curve after BI-82 treatment.

Cell viability

Eight thousand Vero or Huh7-Cre cells were seeded into each well of a white-wall 96-well plate, treated with different concentrations of SMAC mimetics BI-82 and birinapant for 48 hours. Media was aspirated and changed with 100 μl fresh complete DMEM per well, 30 μl CellTiter Glo reagent (Promega) per well was added into each well for 10 min and the luminescence units were read via spectrophotometer (PerkinElmer).

HBV antigens ELISA

Huh7-Cre cells were seeded onto a 24-well plate and pretreated with SMAC mimetics BI-82 and birinapant for 1 hours, then infected with Ad-HBV for 48hpi. Mouse serum samples were either diluted in PBS to measure HBsAg using ELISA kits (Cat#: KH-T-01, KBH) or HBeAg using ELISA kits (Cat#:KH-T-03, KBH) as manufacturer's protocol. For PHH infection, two days before HBV infection, medium changed with fresh 5C medium [5] contains BI-82 or Birinapant with indicated concentration. On Day 0, drugs were washed off and HBV (MOI=100) was added to PHH, gently move the plate in a back-and-forth and side-to-side manner to distribute the virus homogenously. At 18 hpi, HBV was aspirated and rinsed each well gently with Null medium three times. At 3h post first rinse, another three-times-rinse carried on and maintained the infected PHH with 5C medium contains

drugs. Supernatants were collected for ELISA measurement at 7 dpi, and cells were washed by PBS once before DNA extraction (Magen, D3125).

Quantitative real-time PCR

DNA/RNA were extracted with the DNA/RNA extraction kit (Magen, R5111). 2 μg total RNA was used for reverse transcription and qRT-PCR. RNA was reverse transcribed using an All-In-One RT MasterMix (ABM, G492). Real-time PCR (qRT-PCR) analysis of the following genes were carried out using 2xChamQ SYBR qPCR reagents (Vazyme, Q321). qRT-PCR data were normalized to GAPDH, qRT-PCR data of HBV genome DNA and cccDNA normalized to mitochondrial gene level by the Bio-Rad CFX96 system. The primer sets were as follows:

GAPDH-forward primer: 5′-CGGAGTCAACGGATTTGGTCGTAT -3′;

GAPDH- reverse primer: 5′-AGCCTTCTCCATGGTGGTGAAGAC -3′;

cccDNA- forward primer: 5′-CAAGACAGGTTTAAGGAGAC -3′;

cccDNA-reverse primer: 5′- CTGCGGTATTGTGAGGATTC -3′;

3.5kbRNA- forward primer: 5′-GAGTGTGGATTCGCACTCC -3′;

3.5kbRNA- reverse primer: 5′-GAGGCGAGGGAGTTCTTCT -3′;

HBV-DNA- forward primer: 5′-CCGTCTGTGCCTTCTCATCTG -3′;

HBV-DNA-reverse primer: 5′- AGTCCAAGAGTCCTCTTATGTAAGACCTT -3′;

m*α-SMA*- forward primer: 5′-ACTACTGCCGAGCGTGAGAT -3′;

m*α-SMA*-reverse primer: 5′- AAGGTAGACAGCGA GCCAG -3′;

m*Col1a1*- forward primer: 5′-GAAACCCGAGGTATGCTTGA -3′;

m*Col1a1*-reverse primer: 5′- GACCAGGAGGACCAGGAAGT -3′;

m*Col1a2*- forward primer: 5′-TCGTGCCTAGCAACATGCC -3′;

m*Col1a2*-reverse primer: 5′- TTTGTCAGAATACTGAGCAGCAA -3′;

m*MMP2*- forward primer: 5′-TTTGCTCGGGCCTTAAAAGTAT -3′;

m*MMP2*-reverse primer: 5′- CCATCAAACGGGTATCCATCTC -3′;

m*TIMP1*- forward primer: 5′-CTTGGTTCCCTGGCGTACTC -3′;

m*TIMP1*-reverse primer: 5′- ACCTGATCCGTCCACAAACAG -3′;

m*IFN-γ*- forward primer: 5′-CTCTGAGACAATGAACGCTACA -3′;

m*IFN-γ*-reverse primer: 5′- TCTTCCACATCTATGCCACTT -3′;

m*IL-6*- forward primer: 5′-GAGGATACCACTCCCAACAGACC -3′;

m*IL-6*-reverse primer: 5′- AAGTGCATCATCGTTGTTCATACA -3′;

m*IL-10*- forward primer: 5′-TGGCCCAGAAATCAAGGAGC-3′;

m*IL-10*-reverse primer: 5′-CAGCAGACTCAATACACACT-3′;

m*TNF-α*- forward primer: 5′-GTCCCCAAAGGGATGAGAAGTT -3′;

m*TNF-α*-reverse primer: 5′- GTTTGCTACGACGTGGGCTACA -3′;

m*CCL2*- forward primer: 5′-CCCAATGAGTAGGCTGGAGA-3′;

m*CCL2*- reverse primer: 5′-AAAATGGATCCACACCTTGC-3′;

m*CXCL9*- forward primer: 5′- CCAGTAGTGAGAAAGGGTCGC-3′;

m*CXCL9*- reverse primer: 5′- AGGGCTTGGGGCAAATTGTT-3′;

m*TRIM5α*- forward primer: 5′- CCAGTAGTGAGAAAGGGTCGC-3′;

m*TRIM5α*- reverse primer: 5′-ATAGATGAGAAATCCATGGT-3′;

m*ISG15*- forward primer: 5′-GCTGGGACCTGACGGTGA-3';

m*ISG15*- reverse primer: 5′-TGGAGCTGCTCAGGGACAC-3′

h*Mitochondrial*- forward primer: 5′-ACCCACTCCCTCTTAGCCAATATT -3′;

h*Mitochondrial*- reverse primer: 5′-GTAGGGCTAGGCCCACCG -3′;

ZIKV-AS- forward primer: GGTCAGCGTCCTCTCTAATAAACG

ZIKV-AS- reverse primer: GCACCCTAGTGTCCACTTTTTCC

DENV- forward primer: GGCGAAAAACACGCCTTTCA

DENV- reverse primer: TTAATGGTCCTCGTCCCTGC

IAV NP- forward primer: AGGACAAGAGCTCTTGTTCG

IAV NP- forward primer: CTCTTGTGTGCTGGATTCTC

Antibodies

The following antibodies were used for cell cytometry, immunofluorescence staining or immunoblotting: anti-HBcAg (1:200, MA1-7607, Invitrogen), anti-Vinculin (1:1,0000, 4650S,

CST), anti-cIAP1 (1:2,000, 66626-1-Ig, Proteintech), anti-cIAP2 (1:2,000, 24304-1-AP, Proteintech), Anti-Flavivirus Group Antigen Antibody, clone D1-4G2-4-15 (polyclonal mouse, Millipore, MAB10216, 1:1000), anti-XIAP (1:2,000, 10037-1-Ig, Proteintech), peroxidase-conjugated AffiniPure antirabbit antibody (1:20,000, ZB-2301, ZSGB-Bio) and AffiniPure anti-mouse antibody (1:20,000, ZB-2305, ZSGB-Bio), anti-H-2Kb HBV core Tetramer-MGLKFRQL-PE (1:50, TB-M537-1, MBL), Anti-Mouse CD45 FITC (1:300, 07512-50-100, Biogems), anti-mouse CD8 APC (1:300, M10083-11C), Alexa Fluor 488 goat anti-mouse IgG (H+L) (1:1,000; A-11001, Molecular Probes), One Step TUNEL Apoptosis Assay Kit (Beyotime, C1086).

Immunoblotting

Liver leaflets from Alb-Cre mice infected with Ad-HBV and mock infected mice were grounded in liquid nitrogen and then lysed 1% NP40 lysis buffer (50 mM Tris (pH7.5), 100 mM NaCl, 50 mM NaF, 1 mM Na_3VO_3, 1 mM EDTA.Na_2, 1% NP40) on ice. All protein concentrations were determined using the BCA protein quantification methods (Beyotime, P0009), and all protein samples were heated with 5x SDS-PAGE loading buffer (CWBIO, CW0027) for 10 min at 95 °C. Protein samples were separated by 10% SDS–PAGE and electroblotted onto nitrocellulose membranes. After blocking in TBST buffer containing 5% DifcoTM skim milk (BD, 232100) and immunoblot with primary antibody overnight at 4 ℃. Next day, the electroblotted nitrocellulose membranes were washed 3 times, each for 10 min, and incubated with secondary antibody solution for 1 hour at RT to be exposed.

Immunofluorescence

Liver lateral leaflets taken from mock and Ad-HBV infected mice were processed into frozen sections by standard procedures. After being fixed in ice-cold 1% paraformaldehyde (PFA) and permeabilized with 0.5% Tween-20 for 30 min each, sections were treated in antigen recovery buffer (PBS+0.2% Tween-20+0.3 M glycine) for 30 min at room

temperature and washed 3 times with 0.2% Tween-20, then blocked with 5% FBS in 0.2% Tween-20 for 60 min at RT in a humidified chamber. Primary antibody staining was carried out using anti-HBcAg (1:1000, MA1-7607, Invitrogen) with overnight incubation at 4 °C. The next day, slides were washed and incubated with fluorescein-conjugated secondary antibody (1:500, Goat anti-mouse, Invitrogen, R37120, or goat anti-rabbit IgG, 1:500, A-11032, Invitrogen) for 2h at RT. Slides were then washed and covered using a mounting medium with DAPI (ZLI-9557, ZSGB-Bio). Images were taken using a high-performance Slide Scanner (Zeiss Axioscan7), the cell number was counted by ImageJ.

For cell culture staining, eight thousand Vero cells were seeded into each well of a 96-well plate, and next day Vero cells were infected with 1.0 MOI DENV-2 NGC strain and 0.2 MOI ZIKV SZ01 strain for 48 hours. Then, those cells were fixed by directly adding equal volume 4% paraformaldehyde for 15 min at RT. After washing, the cells were permeabilized with 0.2% Triton X-100 (Promega) diluted in PBS for 15 min. Cells were washed with PBS and stained anti-flavivirus group antigen (4G2) (1:1000 dilution) overnight at 4 ℃. Anti-mouse secondary antibody conjugated with Alexa Fluor 488 (Invitrogen, R37120, 1: 1000) was added for a one-hour incubation at RT. After washing three times, the nuclei were stained with DAPI (Invitrogen, 62247, 1:10,000). Images were analyzed by high content microscope (PerkinElmer).

Flow cytometry

Complete blood collected from mice infected with Ad-HBV through lithium heparin BD vacutainer cells, was treated via red cell lysis buffer (RT122, TIANGEN). Lymphocytes were co-stained with anti-mouse CD45 FITC (07512-50-100, Biogems), anti-mouse CD8 APC (M10083-11C, Sungene Biotech) and H-2Kb HBV core tetramer-MGLKFRQL-PE (TB-M537-1, MBL) at RT for 30 min and fixed in 2% paraformaldehyde in PBS in the dark for 30min, washed twice and analyzed by Becton Dickinson LSR Fortessa flow cytometer.

Histopathology

At specified time points, mice were anesthetized with Avertin and perfused with ice-PBS. Liver and lung leaflets were fixed in 4% PFA for two days. PFA-fixed, paraffin-embedded liver and lung sections were stained with hematoxylin and eosin (H&E). For Sirius Red staining, paraffin-embedded sections of the livers were deparaffinized, then hydrated with a series of graded alcohol solutions followed by staining with Sirius Red (GMS80013.3, Genmed). H&E and Sirius Red sections were imaged using a high-performance Slide Scanner (Zeiss Axioscan7).

TUNEL assay

Huh7-Cre cells seeded in 6-well culture plate were transduced with 50 MOI Ad-rcccDNA vector for 3 days. Vero and Hela cells were treated with BI-82 and infected concurrently with 0.1-0.2 MOI flavivirus or IAV for 2 days, respectively. These Ad-rcccDNA transduced cells reseeded in 24-well culture plate containing cell climbing tablets were treated with 10 μM BI-82, Birinapant and DMSO for 2 days. The cells were fixed using 4% paraformaldehyde for 30 min and permeabilized with 0.1% Triton-X for 20 min, and incubated with anti-HBcAg (1:1000, MA1-7607, Invitrogen) or anti-flavivirus group antigen 4G2 or anti-IAV HA antibodies overnight at 4 °C. After being washed 3 times with PBS-T, each sample was stained with 50 μl TUNEL TdT Enzyme working solution (Beyotime, C1086) at 37 ℃ for 60 min and wash 3 times with PBST. The cell climbing tablets were incubated with goat anti-mouse-AF 562nm at RT for 1h. The slides were washed and mounted with Fluoromount-G with DAPI. Images were taken using confocal imaging (ZEISS 780).

Pharmacokinetics analysis by LC-MS/MS

Six male CD-1 mice were orally administrated with BI-82 (217μM/kg) on day 1 and 4 in, and their sera was collected at every day from day 1-day 5 for pharmacokinetics analysis (PK) by LC-MS/MS. An aliquot of 6 μL sera sample, calibration standard, quality control

and dilution quality control, single blank, and double blank sample were added to the 96-well plate respectively. Each sample was quenched with 120 μL of IS1 (6 in 1 internal standard in ACN (Labetalol & tolbutamide & Verapamil & dexamethasone & glyburide & Celecoxib 100 ng/mL for each), double blank sample was quenched with 120 μL of ACN, respectively. And then the mixture was vortex-mixed for 10 min at 800 rpm and centrifuged for 15 min at 3220 × g, 4 °C. An aliquot of 50 μL supernatant was transferred to another clean 96-well plate and centrifuged for 5 min at 3220 × g, 4 °C, then the supernatant was directly injected into Kinetex column (2.6 μ PFP 100 Å 50 × 2.10 mm) for LC-MS/MS analysis, ESI is as negative control. SRM detection of BI-82 is under 467.3 / 391.3 of [M-H]-m/z. UPLC conditions is in mobile phases including A (0.1% FA in water) and B (0.1% FA in CAN). Column temperature is at 45.0 ℃, the sample flow rate is 0.8 mL/min and the sample retention time is 0.95 min.

Quantification and statistical analysis

Statistical analyses were carried out using Prism software (GraphPad). Data are presented as mean ± sem. All experiments were biologically repeated for at least two times. Unpaired two tailed t tests were used to calculate P values. *P<0.05, **P<0.01, ***P<0.001, ****P < 0.0001.

Supplemental Reference

1. Chitalia, V.C. and A.H. Munawar, *A painful lesson from the COVID-19 pandemic: the need for broad-spectrum, host-directed antivirals.* J Transl Med, 2020. **18**(1): p. 390.
2. Mei, M. and X. Tan, *Current Strategies of Antiviral Drug Discovery for COVID-19.* Front Mol Biosci, 2021. **8**: p. 671263.
3. Bai, L., D.C. Smith, and S. Wang, *Small-molecule SMAC mimetics as new cancer therapeutics.* Pharmacol Ther, 2014. **144**(1): p. 82-95.
4. Ladner, S.K., et al., *Inducible expression of human hepatitis B virus (HBV) in stably transfected hepatoblastoma cells: a novel system for screening potential inhibitors of HBV replication.* Antimicrob Agents Chemother, 1997. **41**(8): p. 1715-20.
5. Xiang, C., et al., *Long-term functional maintenance of primary human hepatocytes in vitro.* Science, 2019. **364**(6438): p. 399-402.
6. Petersen, S.L., et al., *Autocrine TNFalpha signaling renders human cancer cells susceptible to Smac-mimetic-induced apoptosis.* Cancer Cell, 2007. **12**(5): p. 445-56.
7. Pache, L., et al., *Pharmacological Activation of Non-canonical NF-kappaB Signaling Activates Latent HIV-1 Reservoirs In Vivo.* Cell Rep Med, 2020. **1**(3): p. 100037.
8. Ebert, G., et al., *Eliminating hepatitis B by antagonizing cellular inhibitors of apoptosis.* Proc Natl Acad Sci U S A, 2015. **112**(18): p. 5803-8.
9. Pache, L., et al., *BIRC2/cIAP1 Is a Negative Regulator of HIV-1 Transcription and Can Be Targeted by Smac Mimetics to Promote Reversal of Viral Latency.* Cell Host Microbe, 2015. **18**(3): p. 345-53.
10. Li, G., et al., *Recombinant covalently closed circular DNA of hepatitis B virus induces long-term viral persistence with chronic hepatitis in a mouse model.* Hepatology, 2018. **67**(1): p. 56-70.
11. Yang, P.L., et al., *Hydrodynamic injection of viral DNA: a mouse model of acute hepatitis B virus infection.* Proc Natl Acad Sci U S A, 2002. **99**(21): p. 13825-30.
12. Maini, M.K., et al., *The role of virus-specific CD8(+) cells in liver damage and viral control during persistent hepatitis B virus infection.* J Exp Med, 2000. **191**(8): p. 1269-80.
13. Molyer, B., A. Kumar, and J.B. Angel, *SMAC Mimetics as Therapeutic Agents in HIV Infection.* Front Immunol, 2021. **12**: p. 780400.
14. Clark, M.P., et al., *Clinical stage drugs targeting inhibitor of apoptosis proteins purge episomal Hepatitis B viral genome in preclinical models.* Cell Death Dis, 2021. **12**(7): p. 641.
15. Zhou, D., et al., *An efficient method of directly cloning chimpanzee adenovirus as a vaccine vector.* Nat Protoc, 2010. **5**(11): p. 1775-1785.

Supplemental Figures

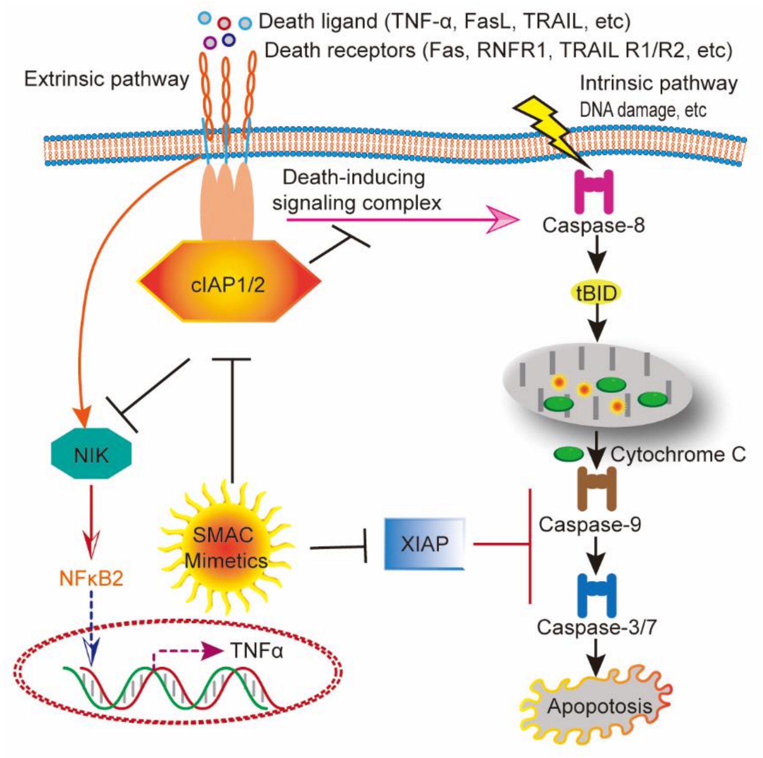

Fig. S1 A cartoon scheme showing the mechanism of action of SMAC Mimetics. TNF-α interaction with TNFR1 and TNFR2 elicits either apoptosis by recruiting different adaptor proteins or NIK/TNF-α inflammation/survival pathway. The extrinsic pathway is triggered by binding of death receptors with death ligands (i.e., TNF-α, FasL, TRIAL, etc.) followed by formation of the death-inducing signaling complex, activation of caspase 8 and transmission of death signals to effector caspase, leading to apoptotic cell death. The intrinsic pathway is induced by a number of factors, including DNA damage to initiate the apoptotic cascade of death signals through interaction with specific downstream mediator of apoptosis. Then the formation of mitochondrial pores and change of membrane permeability release SMACs and cytochrome c into the cytoplasm to facilitate the activation of downstream apoptotic signal (caspase 9, 3, or 7), eventually resulting in apoptosis. Highly expressed IAPs inhibit apoptotic signaling cascades in tumor cells and infected cells with virus. SMAC Mimetics like BI-82, can specifically suppress IAPs protein levels to induce cell apoptosis. TNF, tumor necrosis factor; TRAIL, TNF related apoptosis inducing ligand; tBID, truncated BH3 interacting-domain death agonist; cIAP, cellular

Inhibitors of apoptosis; XIAP, X-linked inhibitor of apoptosis.

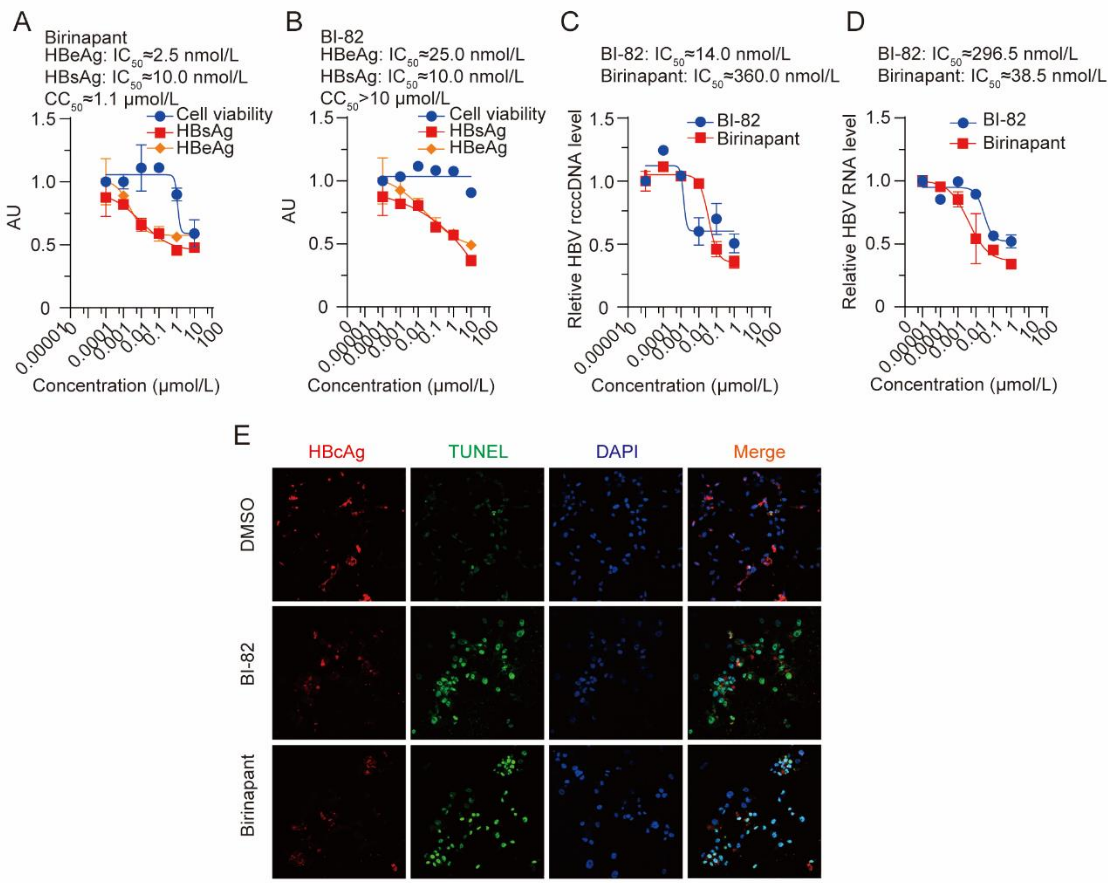

Fig. S2 SMAC Mimetic BI-82 alleviates HBV infection by promoting apoptosis of infected cells in Huh7 cells overexpressing Cre. (A, B) Huh7-Cre cells were treated with different concentration of SMAC Mimetic birinapant (A) or BI-82 (B) and concurrently infected with MOI 50 of Ad-HBV for three days. Supernatant HBe and HBs antigens were quantitated with ELISA. The cell viability was measured with CellTiter Glo after treatment of SMACs at 72 hours, n=3.(C-D) HBV rcccDNA (C) and HBV RNA (D) were measured by quantified PCR after SMACs treatment and Ad-HBV infection in Huh7-Cre at 72 hours, n=3. (E) Cell death and HBcAg expression were determined by TUNEL and anti-HBcAg staining respectively in Ad-rcccDNA transduced Huh7-Cre cells after SMAC Mimetics treatment for 48 h.

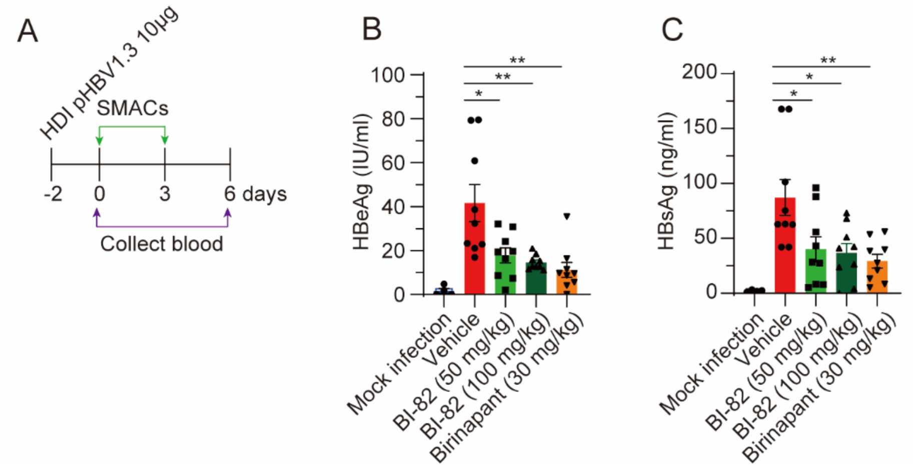

Fig. S3 SMACs alleviates chronic HBV infection in HBV hydrodynamic injection model. (A) Diagram of SMAC Mimetics administration in HBV HDI model, male mice with 8-10 weeks old were injected with 10 μg pHBV1.3 ayw each through hydrodynamic infection. One day later, blood was collected, and mice were grouped with HBV antigen titer quantified by ELISA. At 48 hpi, BI-82 were desolved in 0.5% natrasol, birinapant were desolved in saline and administrated on Day 0 and Day 3 by gavage and intraperitoneal injection (i.p), respectively. On Day 6, blood was collected, and sera HBV antigens were measured by ELISA.(B-C) HBeAg (B) and HBsAg (C) secretion were significantly decreased after SMAC Mimetics administration through ELISA, n=9.

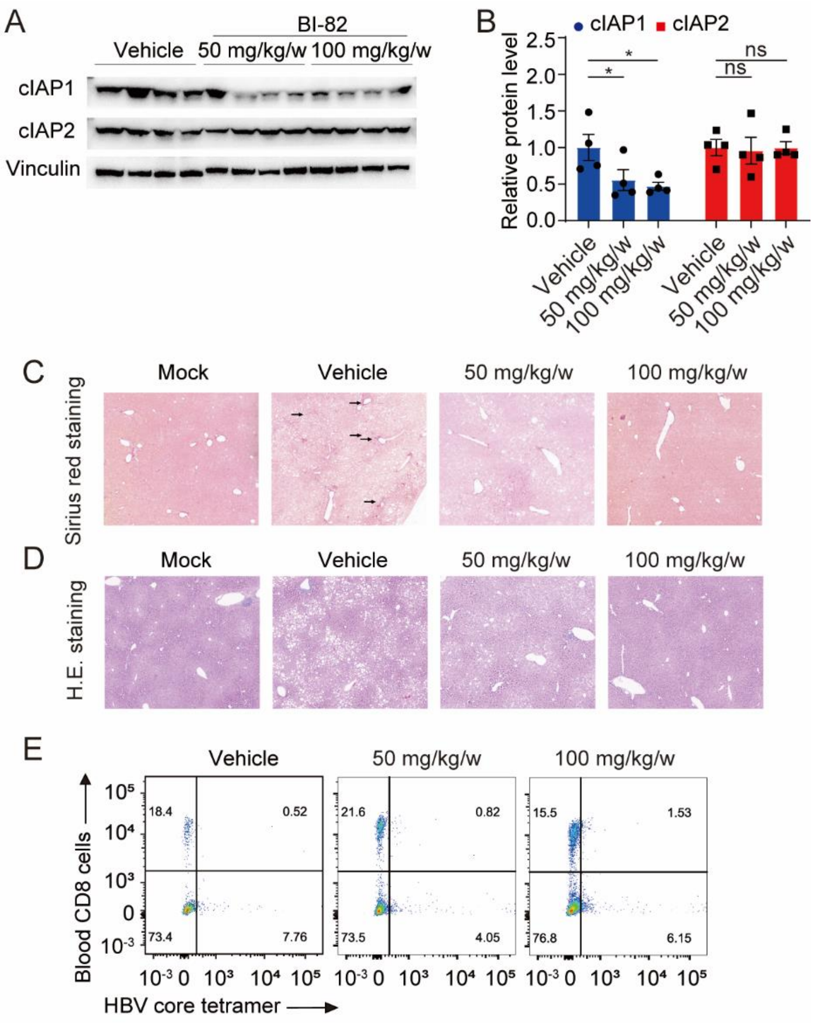

Fig. S4 BI-82 downregulates cIAP1 level, increases HBV-specific CD8⁺ T cells at Week 11 and inhibits liver histopathology at Week 56. (A-B) BI-82 markedly inhibits liver cIAP1 expression by Western blot (C) and protein grayscale quantification (B). The liver is collected on the 7 day after the 10th BI-82 administration. (C) Collagen expression is attenuated in BI-82 treatment groups compared to vehicle treatment group by through Sirius red staining in liver. (D) The liver micro-structure measured by H.E staining is damaged in vehicle treatment group compared to mock infected group. BI-82 treatment groups have much less damage compared to the vehicle group at Week 56 post infection. (E) BI-82 increases HBV core antigen specific $CD8^+$ T cells at week 11.

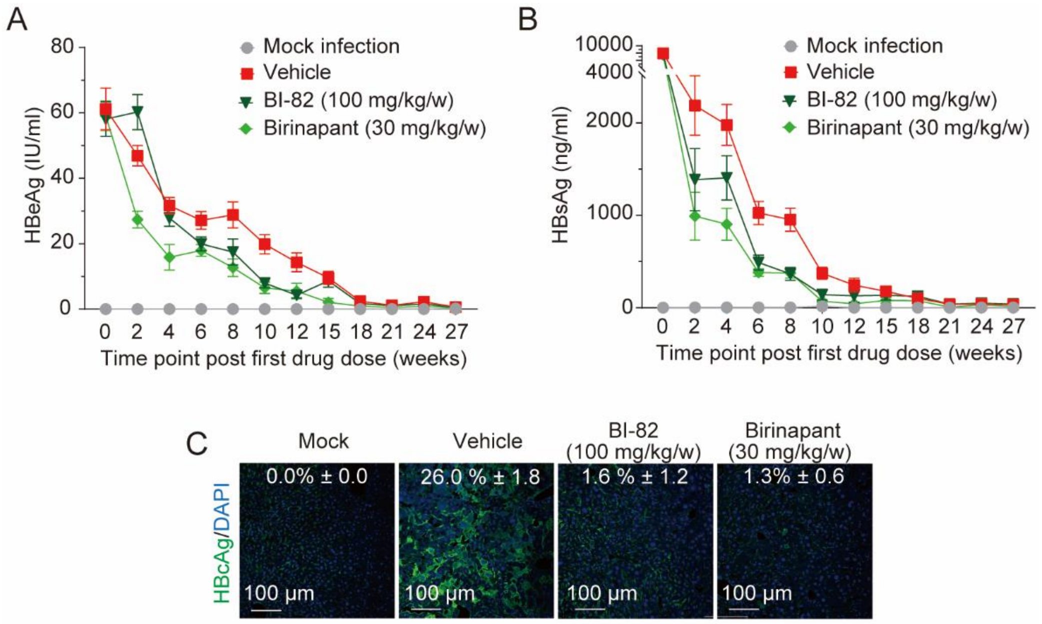

Fig. S5 SMAC Mimetic compounds BI-82 and birinapant inhibit HBV infection in a persistent HBV mouse model. (A-B) SMAC Mimetic compounds BI-82 and birinapant markedly inhibit HBV infection. Every two week, sera HBV antigens were analyzed by ELISA. (C) SMAC Mimetic BI-82 and birinapant inhibit intrahepatic HBV core antigen expression by HBcAg antibody staining.

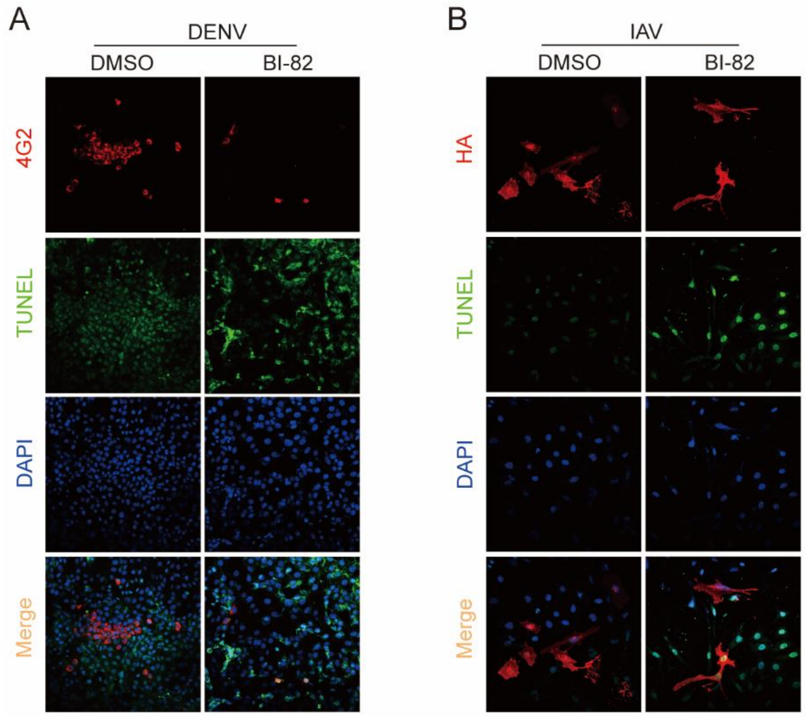

Fig. S6 BI-82 manifests a broad-spectrum antiviral activity through promoting apoptosis of infected cells. (A-B) BI-82 increases apoptosis of DENV (A) and IAV (B) infected cells as shown with TUNEL assay.

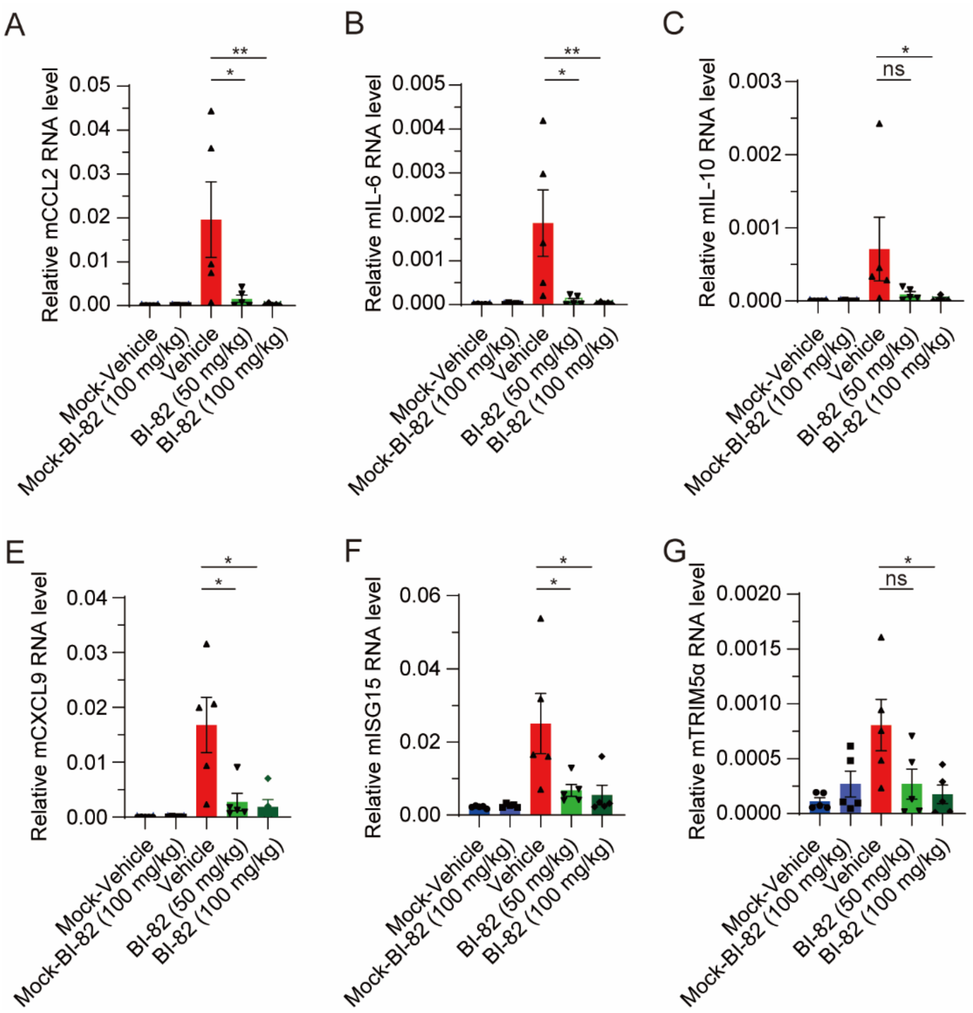

Fig. S7 BI-82 alleviated expression of inflammatory genes and ISGs in lung cells. (A-C) BI-82 alleviated expression of inflammatory genes in the lungs as measured by qRT-PCR. (A-C) BI-82 alleviated expression of ISGs in the lungs as measured by qRT-PCR.
